# Interobserver Variability in the Assessment of Tumor Budding in pT 3/4 Colon Cancer: Improvement by Supporting Immunohistochemistry?

**DOI:** 10.3390/diagnostics10090730

**Published:** 2020-09-21

**Authors:** Benedikt Martin, Patrick Mayr, Regina Ihringer, Eva-Maria Schäfer, Elżbieta Jakubowicz, Matthias Anthuber, Gerhard Schenkirsch, Tina Schaller, Bruno Märkl

**Affiliations:** 1 Institute of Pathology and Molecular Diagnostics, University Medical Center Augsburg, 86156 Augsburg, Germany; patrick.mayr@uk-augsburg.de (P.M.); regina.ihringer@uk-augsburg.de (R.I.); eva-m.schaefer@gmx.net (E.-M.S.); ela@neomed.nazwa.pl (E.J.); tina.schaller@uk-augsburg.de (T.S.); bruno.maerkl@uk-augsburg.de (B.M.); 2 Department of Hematology and Clinical Oncology, University Medical Center Augsburg, 86156 Augsburg, Germany; 3 Department of Visceral Surgery, University Medical Center Augsburg, 86156 Augsburg, Germany; matthias.anthuber@uk-augsburg.de; 4 Tumor Data Management, University Medical Center Augsburg, 86156 Augsburg, Germany; gerhard.schenkirsch@uk-augsburg.de

**Keywords:** colon cancer, tumor budding, interobserver variability, prognostic factor, histology

## Abstract

The prognostic significance of tumor budding in colon cancer is unequivocally documented, and the recommendations of the International Tumor Budding Consensus Conference (ITBCC) are currently the accepted basis for its assessment. Up to now, it is unknown whether the general use of a supporting cytokeratin immunohistochemistry can improve the interobserver variability and prognostic significance. Six investigators with different levels of experience reassessed 229 cases of colon carcinoma (pT3/4, N+/−, M0) with a supporting cytokeratin immunohistochemistry. The results were compared to previous assessments, which have been performed only on H & E. Bd3 was significantly associated with the occurrence of distant metastases according to the assessments of three out of six investigators (*p* < 0.05). Only one single investigator reached significant results concerning the cancer specific survival (*p* = 0.01). The pairwise kappa values range between a poor and moderate level of agreement (range 0.17–0.45; median 0.21). In conclusion, the results show no superiority of the use of an additional cytokeratin immunohistochemistry compared to the conventional analysis on sole H & E slides. Therefore, the general supporting use of a cytokeratin immunohistochemical staining seems to be inadvisable in colon cancer in consideration of necessary resources and costs.

## 1. Introduction

**Figure 1:**
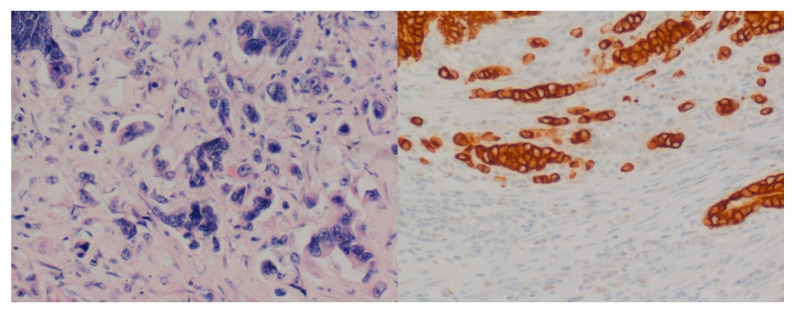
Examples of tumor buds at the invasive front (20×, H & E and cytokeratin staining).

The prognostic significance of tumor budding in colon cancer is unequivocally documented, and the recommendations of the International Tumor Budding Consensus Conference (ITBCC) are the accepted basis for its assessment [1,2,3,4]. Efforts have been made to further enhance the assessment system in regard to interobserver variability and prognostic significance. Especially the assessment on, or with the support of immunohistochemistry (cytokeratin) has been a matter of discussion. Comparative studies to the ITBCC criteria are missing. In this study, six investigators with different levels of experience reassessed 229 cases of pT3/4 colon cancer with the support of cytokeratin staining in the context of an everyday routine setting. The results were analyzed according to the interobserver variability and prognostic significance and compared to prior assessment results, which had been assessed according to the ITBCC. This study is focused on peritumoral tumor budding (PTB; buds at the invasive front, see Figure 1) and does not take intratumoral tumor budding (ITB, buds in the tumor center) into account [1].

## 2. Material and Methods

The present study builds on two previous studies, whose methods have already been published in detail [5,6]. In brief, six investigators (I1, I2, I3, I5, I6, and I7; I4 was not designated to provide a better comparability with the previous study) with different levels of experience (two specialists (I6, and I7), four residents (I1, I2, I3, and I5)) evaluated tumor budding according to the ITBCC method (exception: only one exemplary slide per case) in an approximately everyday routine setting. In the present study, all the investigators reevaluated the same cases under the same conditions, except that cytokeratin staining was available as support. The bud counting for bd grading was performed on H & E. Subsequent classification was divided into three grades: Bd1: 0–4 buds; Bd2: 5–9 buds; and Bd3: 10 or more buds (per 0.785 mm^2^). Afterwards, the investigators counted the buds on the cytokeratin staining for documentation as an additional step. The cytokeratin AE1/AE3 immunostaining was performed according to our routine protocol (immunostainer: Roche Benchmark Ultra (Roche Diagnostics Deutschland GmbH, Mannheim, Germany)); Detection Kit: DAB Opti View IHC (Roche Diagnostics Deutschland GmbH, Mannheim, Germany); antibody: cell marque™, Rocklin, CA, USA, monoclonal mouse antibody; dilution 1:500). Of the 244 cases, 15 (6%) had to be excluded because of the unavailability of the corresponding paraffin block or insufficient immunohistochemical staining result because of improper fixation. Inclusion criteria in brief were primary colon carcinoma (all histologic subtypes); pT3/4, N+/−, M0, R0; resection: 01/02–12/11 at Klinikum Augsburg; survival: >3 months after surgery. We defined a consensus grade, Rcons (sCK) as the grade given by the majority of investigators (with the support of cytokeratin staining), similar to the definition of the consensus grade (Rcons) before. If there were equal numbers of assessments, the higher grade was adopted as Rcons (sCK) (exception: Bd2 was adopted as Rcons (sCK) in the case of a perfectly balanced distribution (ratings: two times Bd1, two times Bd2, and two times Bd3)). Clinical endpoints were overall survival, colon-cancer-specific death, and the occurrence of distant metastasis. Compliance with Ethical Standards: The investigations were carried out following the rules of the Declaration of Helsinki of 1975, revised in 2013. The Institutional Review Board of the Hospital Augsburg reviewed and approved the study protocol (BKF 2017-12, 23 May 2017).

### Statistical Analysis

The Statistical Package for the Social Sciences (SPSS, Chicago, IL, USA), version 24.0, was used for the statistical analyses. The Wilcoxon test was applied for comparisons between continuous and ordinal variables between two dependent groups. Univariate event analyses were carried out according to the Kaplan–Meier method for the assessment of statistical significance (log-rank test). A Cox regression analysis was performed to investigate the independence of univariate-identified risk factors (forward, LR). The hazard ratios (HR) are demonstrated with 95% confidence intervals. The results were considered statistically significant if *p* < 0.05. We calculated kappa statistics for the evaluation of the interobserver variability. Landis and Koch recommend the following interpretation for the kappa values: <0.2 poor; 0.21–0.4 fair; 0.41–0.60 moderate; 0.61–0.80 good; and >0.8 a very good level of agreement [7]. Continuous variables are demonstrated as the mean ± standard deviation (SD), if not otherwise specified.

## 3. Results

### 3.1. Rating Results

The clinicopathological characteristics of the overall patient population (*n* = 244) have been published recently (Table 1) [5]. There was complete agreement among the investigators in 85 (37%) of the included 229 cases concerning the budding grade. The agreement was poor in 50 cases (22%), with a maximum number of only 3 (42 cases, 18%) or 2 (8 cases 3.5%) matching investigators (Table 1). A total of 84 of the 85 cases with complete agreement were classified as Bd1 (one case was classified as Bd3). On average, the maximum number of matching investigators was 4.6, which is slightly lower than in the matched assessments of the previous study (4.8). The kappa values have a range between 0.17 and 0.45 (median 0.21) among the investigators and are slightly higher than in the previous study. The detailed kappa values can be seen in Table 1.

According to Rcons (sCK), 71.6% of the cases were classified as Bd1, 18.8% as Bd2, and 9.6% as Bd3. The budding grades of Rcons (sCK) were significantly higher in comparison to Rcons in the corresponding cases (Rcons: Bd1 82.5%, Bd2 11.8%, Bd3 5.7%, and *p* < 0.001). An overview of the rating results is given in Table 2.

Three of the six investigators counted significantly higher bud numbers and graded higher grades than in the matched cases of the previous study, in which the assessment was only based on H & E (investigator 1 (I1), investigator 5 (I5), investigator 6 (I6) for bud count and grade each <0.001). The mean bud count of all the investigators was 3.6 buds/0.785 mm^2^, counted on the H & E slide, and 9.0 buds/0.785 mm^2^, counted on the cytokeratin slide. In the matched cases of the previous study (only based on H & E), the mean bud counts of all the investigators were 2.6 ± 2.7 buds/0.785 mm^2^ (both comparisons, *p* < 0.001) [5]. The median and interquartile range of the bud counts can be seen in Table 3.

### 3.2. Prognostic Analyses

**Figure 2:**
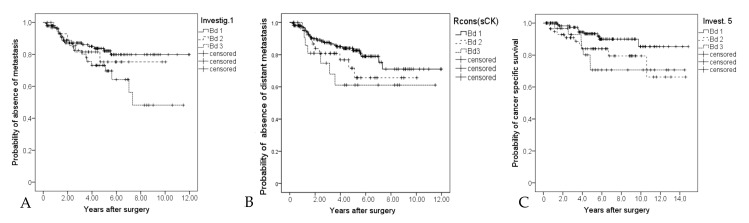
(**A**): Kaplan–Meier curve for the occurrence of distant metastasis (investigator 1) Bd1 vs. Bd3 *p* = 0.049, Bd1 vs. Bd2 and Bd2 vs. Bd3 not significant; (**B**): Kaplan–Meier curve for the occurrence of distant metastasis of the consensus grade (Rcons (aCK)) *p* = 0.15; (**C**): Kaplan–Meier curve for colon-cancer-specific survival (investigator 5) *p* = 0.01.

Bd3 was significantly associated with the occurrence of distant metastasis according to the assessments of I1, I2, and I5 (I1: *p* = 0.049 Bd1 vs. Bd3, Figure 2a; I2: *p* = 0.042 Bd2 vs. Bd3; I5: *p* = 0.005 Bd1 vs. Bd 3; and *p* = 0.029 Bd2 vs. Bd3).

The assessments of Rcons (sCK) are risk stratifying but not statistically significant (Figure 2b). Additionally, the assessments of I5 were significantly associated with colon-cancer-related death (*p* = 0.048 Bd1 vs. Bd2; *p* = 0.023 Bd1 vs. Bd3, Figure 2c). The results of I3 show mainly a significant adverse prognosis of Bd2 cases (overall survival *p* = 0.002 Bd1 vs. Bd2). An overview of all assessment results in terms of prognostic significance is given in Table 2. Multivariate Cox regression (forward, LR) analyses (including: age, pT, pN (+/−)), L, V, grading (low grade/high grade), microsatellite status, and the tumor budding-related results (including the results solely based on H & E from the previous study) was performed for the occurrence of distant metastases. The pT-status (HR 3.4 (1.7–7.1)), MSI-status (instable: HR 0.1 (0.01–0.6)) and the budding grade of I5 (previous study, solely H & E, HR 1.7 (1.1–2.6)) were integrated into the model.

## 4. Discussion

The present study investigates whether the supporting use of a cytokeratin staining reduces the interobserver variability of six investigators with different levels of experience (two specialists, four residents) in the assessment of tumor budding in pT3/4 colon cancer in the context of an everyday routine setting. Furthermore, the influence on the prognostic significance was evaluated. The results indicate no substantial change in the level of observer agreement. The pairwise comparisons between the individual investigators are mainly fair (range: poor to moderate level of agreement) and slightly higher than in the previous (H & E only) assessment, whereas the average of the maximum matching investigators per case is slightly lower (mean 4.6 vs. 4.8) [5]. We could not observe that the interobserver variability of unskilled pathologists was substantially improved by the use of immunohistochemistry, as Kai et al. did [8]. We assume that the advantages and disadvantages of using immunohistochemistry balance each other out. Cytokeratin immunohistochemistry is helpful in the differentiation between fibroblasts, immune cells, and tumor buds, but simultaneously there are potential pitfalls [8,9,10]. Although the counting has been performed on H & E, we assume that the fragmented parts of the tumor glands or parts of cells, especially in areas of inflammation, received higher attention and were thus possibly wrongly classified. Almost all the cases with complete agreement were classified as Bd1. We could already observe in the previous study that a higher grade of tumor budding can often be excluded reliably [6]. The average bud count increased significantly from 2.6 to 3.6 buds per case, with supporting cytokeratin immunohistochemistry (*p* < 0.001) for all investigators. The bud counts and budding grades of three investigators, as well as the budding grades of Rcons (sCK), were significantly higher (all *p* < 0.001) than the assessments, which were solely based on H & E. On average, 9.0 buds/0.785 mm^2^ have been counted on the cytokeratin immunohistochemistry. A 2–4-fold higher number of identifiable buds is consistent with previous findings [10,11]. There are clear differences between the investigators with regard to the number of buds counted (see Table 3). We attribute this primarily to individual differences in the assessment. The difference in the classification did influence the prognostic significance of the budding grades. The prognostic meaning of the assessments of I5 and I6 has been the same, whereas the assessments of I1 and I2 improved in relation to the occurrence of distant metastasis (see Figure 2a,b; I1: *p* = 0.049 Bd1 vs. Bd2; I2: *p* = 0.042 Bd2 vs. Bd3), compared to the previous rating on H & E [5]. Simultaneously, the assessments of I7 diminished their prognostic significance in relation to the occurrence of distant metastasis (*p* = 0.19). Rcons (sCK) did not show statistically significant results, although the Kaplan–Meier curve shows the anticipated split up of Bd1, Bd2, and Bd3 cases (Figure 2b; distant metastasis *p* = 0.15 and colon cancer specific survival *p* = 0.26). In the previous study, Rcons (only H & E) has shown a significant association of the budding grade (Rcons) and the occurrence of distant metastasis (*p* = 0.009) [5]. A multivariate Cox regression analysis (forward, LR), taking the occurrence of distant metastasis as a dependent variable, integrated the budding grade of I5 (solely H & E, HR 1.7 (1.1–2.6)) as an independent risk factor. The results indicate the influence of the assessment method on the prognostic significance, whereby the sum of all the results does not reveal a clear advantage for one method. In conclusion, the results indicate that the general use of supporting cytokeratin staining is not superior to the ITBCC assessment criteria. Other studies comparing cytokeratin(only)-based assessments to H & E-based assessments demonstrated non superiority according to the prognostic value as well [12,13]. Small epithelial structures are straightforward to recognize on cytokeratin, but an elaborate study of experts has shown that the assessment of individual tumor buds using cytokeratin immunohistochemistry has just a moderate level of interobserver agreement, which indicates the issue with cytokeratin staining [14]. Using a supporting cytokeratin staining might be helpful in special situations, but a limitation of this study is that there was no standardized recording of qualitative factors (for example, inflammation). For this reason, no statements can be made on this subject. Apart from the use of cytokeratin immunohistochemistry, the number of alternative methods is limited. Besides consultation and extending the assessment area, digital methods might be the most promising approach to support the assessment [5,15]. A semiautomatic approach has shown convincing results, whereas more advanced algorithms are being explored as well [16,17,18]. In particular, machine learning techniques will substantially contribute to solve the task, but the question of the “right” budding grade might then possibly be obsolete, too [19].

## 5. Conclusions

The supporting use of cytokeratin immunohistochemical staining has not improved the interobserver variability in the evaluation of tumor budding in pT3/4 colon cancer among investigators of different levels of experience. Irrespective of this, its usage significantly increased the bud counts and bd grades and influenced the prognostic significance. Overall, the results show that a general supportive use of cytokeratin immunohistochemical staining seems to be inadvisable in colon cancer, considering the associated resources and costs required for the technique and given the lack of convincing advantages in its results.

## Authors Contributions

All the authors revised the article critically, contributed with reflective improvements, and approved the final version. B.M. (Bruno Märkl), B.M. (Benedikt Martin), and E.-M.S. contributed to the study conception and design. B.M. (Benedikt Martin), E.J., P.M., R.I., T.S., and B.M. (Bruno Märkl) contributed to the histopathologic analyzation and interpretation. E.-M.S., G.S., M.A., and B.M. (Benedikt Martin) contributed to the data acquisition. B.M. (Bruno Märkl), B.M. (Benedikt Martin), and P.M. contributed to the data analysis and interpretation. All authors have read and agreed to the published version of the manuscript.

## Figures and Tables

**Table 1:** Agreements among the investigators and kappa values for paired comparisons among the investigators and the consensus rating, Rcons (sCK).

Agreement	6 of 6	5 of 6	4 of 6	3 of 6	2 of 6		
*n*	85	34	60	42	8		
%	37%	15%	26%	18%	4%		
Kappa values							
	R1	R2	R3	R5	R6	R7	Rcons (sCK)
I1	x	0.27	0.18	0.29	0.31	0.45	0.5
I2	0.27	x	0.17	0.25	0.2	0.29	0.39
I3	0.18	0.17	x	0.14	0.19	0.22	0.36
I5	0.29	0.25	0.14	x	0.21	0.39	0.53
I6	0.31	0.2	0.19	0.21	x	0.35	0.54
I7	0.45	0.29	0.22	0.39	0.35	x	0.67
Rcons (sCK)	0.5	0.39	0.36	0.53	0.54	0.67	x

**Table 2:** Overview of the rating results in terms of the prognostic significance.

	Mean Bud Count		Bd1 ^1^		Bd2 ^1^		Bd3 ^1^		*p*
	H & E ^1^	CK							
	0.785 mm^2^	0.785 mm^2^	*n*	%	*n*	%	*n*	%	
Rcons (sCK)			164		43		22		
Death			60	37%	21	49%	12	55%	0.22
Cancer-specific death			17	10%	5	12%	5	23%	0.26
Distant metastasis			27	16%	10	23%	7	32%	0.15
Investigator 1	4.9 ± 5.8	11.2 ± 13.5	144		29		56		
Death			56	39%	13	45%	24	43%	0.72
Cancer-specific death			12	8%	5	17%	10	18%	0.22
Distant metastasis			21	15%	6	21%	17	30%	0.15/0.05 *
Investigator 2	2.9 ± 3.1	7.0 ± 5.7	154		52		23		
Death			64	42%	17	33%	12	52%	0.20
Cancer-specific death			17	11%	4	8%	6	26%	**0.02**
Distant metastasis			31	20%	6	12%	7	30%	**0.13/0.04 °**
Investigator 3	2.8 ± 3.4	6.6 ± 6.7	176		48		5		
Death			62	35%	29	60%	2	40%	**0.01**
Cancer-specific death			19	11%	7	15%	1	20%	0.48
Distant metastasis			28	16%	16	33%	0	0%	**0.01**
Investigator 5	4.1 ± 4.4	11.1 ± 7.5	138		59		32		
Death			50	36%	28	47%	15	47%	0.51
Cancer-specific death			10	7%	10	17%	7	22%	**0.05**
Distant metastasis			21	15%	11	19%	12	38%	**0.01**
Investigator 6	2.7 ± 2.9	7.7 ± 5.8	167		48		14		
Death			68	41%	19	40%	6	43%	0.94
Cancer-specific death			18	11%	8	17%	1	7%	0.43
Distant metastasis			32	19%	10	21%	2	14%	0.71
Investigator 7	4.2 ± 9.5	10.1 ± 13.9	162		30		37		
Death			61	38%	15	50%	17	46%	0.30
Cancer-specific death			16	10%	6	20%	5	14%	0.18
Distant metastasis			28	17%	9	30%	7	19%	0.19

^1^ the bud counting for bd grading was performed on H & E; a cytokeratin staining was available as support for the identification of buds; *p*: values for Kaplan–Meier analyses; pairwise comparisons: * Bd1 vs. Bd3 *p* = 0.049; ° Bd2 vs. Bd3 *p* = 0.042. The bold numbers are statistically significant.

**Table 3:** Overview of the median, minimum, maximum, and interquartile.

Range (Q1, Q3) of Bud Counts										
	H & E (0.785 mm^2^)					CK (0.785 mm^2^)				
	Min	Q1	Median	Q3	Max	Min	Q1	Median	Q3	Max
Investigator 1	0	0	2	9	33	0	2	7	17	76
Investigator 2	0	0	3	4	12	0	3	6	10	20
Investigator 3	0	0	2	4	17	0	0	5	10	25
Investigator 5	0	1	2	6	22	0	5	10	17	26
Investigator 6	0	0	2	4	12	0	4	6	12	33
Investigator 7	0	0	0	5	83	0	0	4	17	83

The bud counting for bd grading was performed on H & E; cytokeratin staining was available as support for the identification of buds.
